# Sweet Puppies and Cute Babies: Perceptual Adaptation to Babyfacedness Transfers across Species

**DOI:** 10.1371/journal.pone.0058248

**Published:** 2013-03-13

**Authors:** Jessika Golle, Stephanie Lisibach, Fred W. Mast, Janek S. Lobmaier

**Affiliations:** 1 Department of Psychology, University of Bern, Bern, Switzerland; 2 Center for Cognition, Learning, and Memory, University of Bern, Bern, Switzerland; University of Minnesota, United States of America

## Abstract

Infant faces are very salient stimuli. The Kindchenschema describes specific features that characterize a cute infant face. In this study we used a visual adaptation paradigm to investigate the universality of the perceptual properties of the Kindchenschema. In Experiment 1, twenty-four participants adapted to cute and less cute human infant faces and in Experiment 2, twenty-four new participants adapted to cute and less cute faces of puppy dogs. In both experiments the task was to assess the cuteness of subsequently presented human infant faces. The results revealed cuteness after-effects for human infant faces in both adaptation conditions, suggesting a common mechanism coding cuteness in human and non-human faces. This study provides experimental evidence for the universality of the well-described concept of the Kindchenschema.

## Introduction

Immediately after birth, infants of most species rely largely on parental care-taking behavior, such as protecting and feeding. Care-taking behavior is crucial for the survival of the next generation and thus secures the survival of the whole species. Characteristics inherent in infant faces facilitate such behavior. For example, Konrad Lorenz (1943) [1] described the *Kindchenschema* as an innate releasing mechanism for care-taking behavior. According to Lorenz, the Kindchenschema works as a trigger for instinctive behavior such as taking a child into one’s arms. The Kindchenschema is characterized by pedomorphic features such as a relatively large head compared to the size of the body, a relatively big cranium compared to the facial bones, large eyes that lie below the horizontal midline of the skull, a soft-elastic surface texture, and round and protruding cheeks. Infants that conform to the Kindchenschema are commonly described as cute and evoke positive affective approach behavior.

Several investigations found empirical support for Lorenz’ observations [2], [3]–[8]. Sternglanz and colleagues (1977) [4] parametrically varied eye height, eye width, iris size, and vertical feature position and had participants rate the attractiveness of these faces. They found that especially a large forehead associated with a small chin and large eyes make a baby face look cute. Glocker and colleagues (2009) [8] varied the width and the length of the whole face and the size of the forehead, the eyes, the nose, and the mouth of infant faces. A round face, a high forehead, big eyes, a small nose, and a small mouth were defined as “high” Kindchenschema features. They found that cuteness ratings were positively related to the extent of the Kindchenschema. Furthermore, the level of Kindchenschema correlates with positive attributions such as cuteness, warmth, and honesty [9], and has a positive emotional influence on mother-child interaction. Mothers of cute babies show more affectionate interactions compared to mothers with relatively less cute babies [10].

Lorenz (1943) [1] claimed that the Kindchenschema is found in various species (e.g., humans, ducks, hares, tigers, lions, and dogs). To date, there is only one recent study that experimentally scrutinized the universality of the Kinschenschema. Little (2012) [11] systematically varied the proportion of adult- and babyfacedness in cats, human babies, and adults by approximating their shape to human adult and baby composite faces. The results of a cuteness-rating task revealed that both cat and human faces were assessed cuter when approximated towards the shape of a human baby face compared to when approximated towards the shape of a human adult face. Thus, facial shape adopted from human infants (small jaw, large forehead) influenced the cuteness evaluation of cat faces. Our study went beyond the influence of facial shape on cuteness perception. We used a perceptual adaptation paradigm to test whether the processing of cuteness is species-specific or whether a generic mechanism underlies the perception of cuteness.

Perceptual adaptation is characterized by a change in the perception of stimuli after prolonged exposure to a specific stimulus [12]. As a consequence subsequent perception is biased. Such a bias is termed after-effect and can occur for different perception modalities. Visual after-effects have been demonstrated for low-level features such as color or motion, as well as for rather complex stimuli such as faces. After-effects that are independent of changes in low-level features are referred to as high-level after-effects. Their occurrence indicates that the adaptation involves higher visual processing areas. Visual high-level after-effects have been reported for many different facial attributes: identity [13], sex [14], eye gaze direction [15]–[17], emotional expression [14], [18], [19], attractiveness [20], ethnicity [14] and age [21]. Valentine’s (1991) [22] norm-based coding model of the representation of faces is often used to explain the visual after-effects found for facial attributes. This model suggests that faces are represented in a multi-dimensional face space. In the center of this face space the average of all faces (prototype) is stored and all possible dimensions that describe a face (e.g., age, race etc.) are arranged like a fan around the center and cross in the prototype. An after-effect can be explained by a recalibration of the prototype [23]; the position of the average face on a dimension trajectory (e.g., age) temporarily shifts towards the adapting condition. Subsequently presented faces are then assessed in contrast to the “new” average: The same face is seen as older after the exposure to young faces compared to after the exposure to old faces [21].

So far, all studies reporting high-level after-effects have focused on the processing of facial attributes in adult faces. However, no study has yet investigated how infant faces are situated within the multi-dimensional face space. At least some dimensions on which a face is represented seem to vary between infant and adult faces. For example, although cuteness and attractiveness both represent beauty, attractiveness is a dimension that more appropriately describes adult faces because it has a sexual connotation. In contrast, cuteness applies specifically to infant faces because it triggers care-taking behavior. Indeed, previous studies showed that the Kindchenschema positively influences careful behavior [24], [25]. Participants were better in a fine motor dexterity task when they previously saw infant faces compared to adult faces. Furthermore, likeability ratings of cute infants were higher and the willingness to look at them was stronger compared to faces of adult people [26]. While there is one study that reported high-level attractiveness after-effects [20] no study has yet examined high-level after-effects for facial cuteness.

Here we aim at establishing whether there are cuteness after-effects for infant faces and if so, whether these after-effects transfer across species. In Experiment 1 participants adapted to cute and less cute human infant faces and subsequently assessed the cuteness of human infant faces. In Experiment 2 participants adapted to cute and less cute puppy dogs, and then assessed the cuteness of human infants, to scrutinize a possible transfer of cuteness after-effects across species. So far, only one study investigated facial after-effects for faces of different species [27]. They found a species-specific after-effect for eye-spacing in human and monkey faces. The authors interpreted this finding as evidence for functionally distinct neural populations coding faces of different species and for discrete representations of these faces in the human brain. While these findings speak against a common coding of cuteness for different species, Lorenz (1943) [1] emphasized the universality of the Kindchenschema. According to his observations, cuteness may be represented by a species-unspecific coding mechanism, since infant faces of different species are characterized by the same facial features (e.g. large eyes, big cranium, etc). Following this universal concept of the Kindchenschema, facial characteristics that constitute a cute face should apply indiscriminately for different groups of vertebrates such as mammals or birds. Using an adaptation paradigm allows us to investigate whether infant cuteness perception of different species involves a common processing mechanism. If the Kindchenschema is indeed universal, we expect the cuteness after-effect to transfer from faces of puppy dogs to human infant faces.

We conducted two experiments to investigate whether high-level cuteness after-effects occur and whether these effects transfer across species. In order to test the existence of cuteness after-effects participants adapted to cute and less cute human infant faces and assessed the cuteness of subsequently presented human infant faces (Experiment 1). We then investigated whether cuteness after-effects transfer across species (Experiment 2). Specifically, we examined whether the exposure to cute and less cute puppy faces influences the evaluation of cuteness in subsequently presented human infant faces.

## Experiment 1

The aim of Experiment 1 was to ascertain whether high-level cuteness after-effects occur for human infant faces. Participants adapted to cute and less cute human infant faces and assessed the cuteness of subsequently presented human infant faces. The existence of a cuteness after-effect would be manifest in differing cuteness ratings, depending on whether participants adapted to cute or less cute infant faces.

### Methods

#### Participants

Twenty-four (12 female, 12 male) undergraduate students of the University of Bern ranging in age between 20 and 34 (*M* = 23.8, *SD* = 3.4) participated in this experiment. All had normal or corrected-to-normal eyesight as indicated by self-report. All participants gave written informed consent and received either course credits or a snack in return for their participation. The research was approved by the ethics committee of the Faculty of Human Sciences of the University of Bern and conformed with the “Ethical Principles of Psychologists and Code of Conduct” of the American Psychological Association (2002).

#### Stimuli

The stimuli consisted of colored, front-view faces of Caucasian infants showing a neutral facial expression, collected from different web pages. First, we identified cute and less cute stimuli. For this, 10 participants (5 female) rated the cuteness of 78 human infant faces on a 7-point rating scale (1 = not at all cute to 7 = very cute, Cronbachs Alpha = 0.91). Thirteen faces that were rated equal or greater than 5 were taken as cute stimuli, 13 faces that were rated less than 3 were taken as less cute stimuli. The 5 faces that were rated cutest (*M* = 5.8, *SD* = 0.77) and the 5 faces that received the lowest cuteness ratings (*M* = 2.3, *SD* = 0.74) were used as adaptor stimuli. A paired sample *t*-test revealed that the mean ratings for the cute und less cute babies significantly differed from each other, *t*(9) = −9.27, *p*<.001. The remaining faces were individually paired (8 cute with 8 less cute infant faces) and each pair was transformed in shape and color using PsychoMorph computer graphics software [28], [29]. The test stimuli consisted of transformations representing cute/uncute proportions of 20%/80%, 40%/60%, 60%/40% and 80%/20%, totaling in 32 test stimuli [8 (transformation pairs)×4 (20%, 40%, 60% and 80% proportion of cuteness)]. The adaptor stimuli had a size of 1199×1210 mm and a resolution of 72 dpi. Participants were seated 60 cm in front of a 23″ computer screen with a resolution of 1920×1200 pixel; thus, the stimuli subtended a visual angle of 5.75°×9.34°. The test stimuli measured 741×748 mm, had a resolution of 72 dpi, and subtended a visual angle of 3.55°×5.78°. The size of the adaptor and test stimuli differed to ensure that potentially observed facial cuteness after-effects were not based on low-level stimulus characteristics [13], [16]. There was no overlap between the identities of adaptor and test stimuli, in order to rule out repetition effects [30].

#### Procedure

We used the same procedure as Schweinberger and colleagues (2010) [21]. Each participant underwent two experimental conditions. In one condition they adapted to cute, in the other to less cute human infant faces. The order of experimental conditions was counterbalanced across participants. Each experimental condition consisted of 3 phases: pre-adaptation, adaptation and post-adaptation. In the *pre-adaptation phase* all test stimuli were randomly presented and the task was to rate the cuteness of these faces on a QWERTZ computer keyboard using the keys “S”, “D”, “V”, “N”, “K” and “L” on which we added stickers that were marked with the values 1 to 6 (reflecting the cuteness levels, 1 = uncute, 6 = cute). The participants were asked to use the index finger, middle finger and ring finger of each hand to enable spontaneous and fast reaction. Prior to the experiment proper the participants familiarized with the task in 20 practice trials. The stimuli used in the practice trials were not used in the subsequent phases. Each trial started with a fixation cross for 500 ms and was followed by a face which was presented for 1000 ms. After the presentation of the test stimulus the participants had 1500 ms to give an answer. Trials were separated by a black screen (1000 ms). Feedback was given only if the participant responded too slowly or pressed a “wrong” key (different than the marked keys). Each stimulus appeared twice, resulting in a total of 64 ratings. In the *adaptation phase* 5 adaptor faces were presented for 4000 ms each and the participants were instructed to attentively look at the faces. Each adaptor face was shown twice in a random order. In the following *post-adaptation phase*, all test stimuli were again presented and participants were asked to evaluate their cuteness. In contrast to the pre-adaptation phase, two top-up adaptor faces were presented for 2000 ms each before a test stimulus was shown. Between the adaptor faces and the test stimulus a question mark (500 ms) informed the participants that they were expected to rate the cuteness of the following face. As in the other phases, the order in which the faces were shown was randomized.

### Results and Discussion

We calculated an analysis of variance (ANOVA) with adaptation condition (uncute, cute) and morph level (20%, 40%, 60% and 80% of the cute face) as within subject factors and participant sex as between subject factor. The dependent variable was the mean evaluation of cuteness in the post-adaptation phase (see also [21]). Due to omitted or delayed responses we excluded less than 2.6% of all trials. The Huynh-Feldt epsilon correction for heterogeneity of covariances [31] was used when sphericity could not be assumed. For post-hoc pairwise comparisons we used the Bonferroni correction.

There was a significant main effect of adaptation condition, *F*(1, 22) = 38.33, *p*<.001, *η_p_^2^* = .64. After adapting to less cute infant faces the faces in the post-adaptation phase were assessed cuter (*M* = 4.03, *SE* = 0.13) compared to after adapting to cute faces (*M* = 3.29, *SE* = 0.13). The effect of morph level was also significant, *F*(1.3, 29.4) = 39.56, *p*<.001, *η_p_^2^* = .64. Post-hoc pairwise comparisons revealed that faces that were morphed to look cuter were assessed as being cuter. The effect of participant sex was not significant. Neither the main effect nor the interaction with adaptation condition or morph level reached statistical significance (all *p*’s >.149).

These results provide clear evidence for the existence of cuteness after-effects in human infant faces. Although high-level after-effects have been reported for attractiveness of adult faces, we are the first to report similar after-effects for infant faces. In terms of the norm-based coding model of the representation of faces [22] the findings of Experiment 1 suggest that cuteness is a valid dimension for infant faces.

After having established cuteness after-effects in infant faces, the next step is to investigate whether this dimension is species-specific (i.e. applies only for human infants) or whether the same concept underlies the cuteness assessment of faces of different species.

## Experiment 2

Experiment 2 examined whether the cuteness after-effects found in Experiment 1 transfer across species. Participants adapted to cute and less cute puppy dogs and subsequently rated the cuteness of human infant faces. If cuteness after-effects indeed transfer across species, this would be strong evidence for Lorenz’ (1943) [1] claim that the Kindchenschema is universal.

### Methods

#### Participants

Twenty-four (12 female, 12 male) undergraduate students of the University of Bern aged between 21 and 28 (*M* = 23.6, *SD* = 1.8) participated in this experiment. All had normal or corrected-to-normal eyesight as indicated by self-report. All participants gave written informed consent and received either course credits or a snack in return for their participation. The research was approved by the ethics committee of the Faculty of Human Sciences of the University of Bern and conformed with the “Ethical Principles of Psychologists and Code of Conduct” of the American Psychological Association (2002). None of these participants had participated in Experiment 1.

##### Stimuli

In Experiment 2 the adaptor stimuli were front-view puppy faces of different dog breeds. Cute and less cute puppy faces were identified in a previous cuteness rating. Ten participants (5 male) rated the cuteness of 69 puppy faces on a 7-point rating scale (1 = not at all cute to 7 = very cute, Cronbachs Alpha = .90). The 5 faces that were rated cutest (*M* = 5.62, *SD* = 0.72) and the 5 faces that received the lowest cuteness ratings (*M* = 2.36, *SD* = 0.86) were used as adaptor stimuli. A paired sample *t*-test revealed that the mean ratings for the cute und less cute puppies significantly differed from each other, *t*(9) = −10.57, *p*<.001. The test stimuli were the same as in Experiment 1.

#### Procedure

The procedure was the same as in Experiment 1, with the sole exception that cute and less cute puppy faces were used as adaptor stimuli.

### Results and Discussion

The data were analyzed as in Experiment 1. Less than 4.5% of trials were excluded because the participants did not react or responded too slowly. We found a significant main effect of adaptation condition, *F*(1, 22) = 6.32, *p* = .020, *η_p_^2^* = .22. After adapting to less cute puppies, human infant faces were assessed as being cuter (*M* = 3.36, *SE* = 0.14) compared to after adapting to cute puppies (*M* = 3.21, *SE* = 0.10). The main effect of morph level was also significant, *F*(2, 46.2) = 121.73, *p*<.001, *η_p_^2^* = .84, reflecting that faces that were manipulated to be cute were evaluated cuter than the faces that were manipulated to be less cute. Neither the main effect of participant sex nor the interaction between participant sex and adaptation condition reached statistical significance (all *p*’s >.352). The factor participant sex interacted with the factor morph level, *F*(2.6, 57.1) = 7.65, *p*<.001, *η_p_* ^2^ = .26, indicating that female participants assessed 80% cute infants cuter than male participants. For all the remaining morph levels (20%, 40%, 60%) there were no differences between male and female observers.

In Experiment 2 we found that adapting to cute or less cute faces of puppy dogs influenced the cuteness assessment of human infant faces. After adapting to cute puppy dogs, human infant faces were evaluated to be less cute, while the same human infants were rated to be cuter after adapting to less cute puppies. We note that the after-effect was smaller when adaptor and test faces were of different species. In the first experiment the size of the effect was larger (*η_p_* ^2^ = .63) compared to the effect in the second experiment (*η_p_* ^2^ = .22). This however does not take away from our claim that there is a common coding mechanism for cuteness.

## General Discussion

In the present study we used a visual adaptation paradigm to investigate high-level cuteness after-effects in human infant faces and whether these after-effects transfer across species. In Experiment 1 participants adapted to cute and less cute human infant faces and assessed the cuteness of subsequently presented human infant faces. In Experiment 2 participants adapted to cute and less cute puppy faces and evaluated the same human infant faces used in Experiment 1. The results of both experiments revealed that after adapting to less cute faces, human infant faces were evaluated as being cuter, whereas the same faces were rated less cute after adapting to cute faces.

The results of Experiment 1 are the first demonstration of high-level cuteness after-effects in infant faces: the perception of cuteness changes as a result of the exposure to cute, compared to less cute, faces. In terms of the norm-based coding model for faces [22], exposure to cute or less cute faces leads to a shift of the average face towards the adapted condition and thus leads to a new reference to assess the cuteness of infant faces. While previous studies on visual high-level after-effects focused on facial characteristics in adult faces (e.g., age, attractiveness, sex) we are the first to investigate after-effects for a facial attribute that is particular in infant faces. Cuteness in infant faces is presumably important for the survival of the species as it allegedly triggers care-taking behavior, which involves feeding and protecting the offspring.

Our most interesting finding is that the effects of facial cuteness adaptation transfer across species. Naturally, the within-species after-effects were larger compared to the across-species after-effects. Nevertheless, we found similar after-effects for human infant faces after participants were exposed to cute and less cute faces of puppy dogs. These findings support Konrad Lorenz’ (1943) [1] claim of a species-unspecific Kindchenschema. According to Lorenz, a cute infant face is determined by specific pedomorphic characteristics which are present in many different species (e.g., dogs, tigers etc.). Our data suggest a common mechanism that codes the cuteness of human and non-human infant faces. This result can be taken as experimental evidence for the universality of the Kindchenschema. Although we used only puppy dogs and human infant faces, we would expect to find similar transfer effects of facial cuteness adaptation for various species which rely on brood care.

A recent study by Sato et al. (2012) [32] reported empirical evidence for across-species preferences for infant over adult faces. They found longer looking durations in adult female Japanese macaques for infant compared to adult faces of Campbell's monkeys. In addition, the study by Little (2012) [11] found that when cat and human faces were assimilated to the shape of human infant faces, both cats and humans were assessed cuter than when they were assimilated to the adult-like shape. In the present study we went beyond the impact of facial shape and provide evidence for a common processing mechanism that underlies cuteness perception across species. Employing a visual adaptation paradigm we found that the adaptation to facial cuteness in puppy faces affected the cuteness perception in human infant faces. While our findings cannot elucidate which features actually make a face cute, we could show that cuteness perception of different species involves common processing mechanisms. This can be taken as direct empirical evidence in favor of a universal processing mechanism of facial cuteness.

Lorenz described the Kindchenschema as an innate releasing mechanism for care-taking behavior in that a cute infant will extract more rather than less care from its primary caretaker. Our present findings suggest that this is not only true for infants of the own species, but also for infants of a different species. Thus, from the viewpoint of the newborn, being sufficiently cute is the first step towards a pivotal commitment: protecting and feeding the immature [33]. Our findings suggest that human beings have a general instinct to take care of newborns, be it of the same or of another species. In line with this notion, previous studies [24], [25] reported a positive influence of kitten and puppy faces on careful behavior in human subjects. The participants showed a better performance in a fine motor dexterity task when they previously saw kitten and puppy faces compared to faces of adult dogs and cats. While this instinct might not have a proximate benefit for the caregiver, it is advantageous for the infant.

The results of this study are not in line with the findings of Little and colleagues (2008) [27] who studied after-effects after adapting to wide or narrow eye-spacing in faces of monkeys and humans. They claim that faces of different species are coded by functionally distinct neural populations and are discretely represented within the human brain. Although eye-spacing in adult faces might be coded discretely for different species, our findings suggest that infant cuteness is coded by a generic mechanism that does not differentiate between species.

Taken together, the present study is the first to report a facial cuteness after-effect in human infant faces. More interestingly, we found that the cuteness after-effect transfers from puppy dogs to human infant faces: adaptation to cute and less cute puppy faces affected the perception of facial cuteness in human infant faces. This can be taken as evidence for a common mechanism coding the cuteness of faces. To our best of knowledge we are the first to experimentally confirm Lorenz’ (1943) [1] claim that a universal mechanism underlies the processing of infant cuteness.
